# The Plastid Genome of the Red Macroalga *Grateloupia taiwanensis* **(**Halymeniaceae**)**

**DOI:** 10.1371/journal.pone.0068246

**Published:** 2013-07-19

**Authors:** Michael S. DePriest, Debashish Bhattacharya, Juan M. López-Bautista

**Affiliations:** 1 Department of Biological Sciences, The University of Alabama, Tuscaloosa, Alabama, United States of America; 2 Department of Ecology, Evolution, and Natural Resources, Rutgers University, New Jersey, United States of America; University of Melbourne, Australia

## Abstract

The complete plastid genome sequence of the red macroalga *Grateloupia taiwanensis* S.-M.Lin & H.-Y.Liang (Halymeniaceae, Rhodophyta) is presented here. Comprising 191,270 bp, the circular DNA contains 233 protein-coding genes and 29 tRNA sequences. In addition, several genes previously unknown to red algal plastids are present in the genome of *G. taiwanensis*. The plastid genomes from *G. taiwanensis* and another florideophyte, *Gracilaria tenuistipitata* var. *liui*, are very similar in sequence and share significant synteny. In contrast, less synteny is shared between *G. taiwanensis* and the plastid genome representatives of Bangiophyceae and Cyanidiophyceae. Nevertheless, the gene content of all six red algal plastid genomes here studied is highly conserved, and a large core repertoire of plastid genes can be discerned in Rhodophyta.

## Introduction

The red algae (division Rhodophyta) comprise over 6,300 species [1] of mostly multicellular, marine, photosynthetic organisms. Along with Viridiplantae (green algae and higher plants) and Glaucophyta, Rhodophyta is one of the three lineages of eukaryotes originating from primary endosymbiosis of an ancient cyanobacterium, forming the supergroup Plantae *sensu lato*. The monophyly of Plantae *s.l*. is well supported in several analyses [2][3[4]. Subsequent secondary endosymbioses have occurred, resulting in a great diversity of plastid-bearing eukaryotes throughout the tree of life. The chlorarachniophytes and euglenoids separately acquired green algal endosymbionts, whereas the numerous “brown” lineages (including haptophytes, cryptophytes, stramenopiles, and alveolates) acquired red algal endosymbionts. It remains unclear, however, at which point (or points) in evolutionary history the acquisition of those red algal plastids took place, and several hypotheses have been suggested to explain the pattern, which have been tested and supported to varying degrees [5]. However, it is clear that additional data collection and analysis are needed for both the hosts and endosymbionts in this partnership, that is, for brown algal lineages and the red algae from which their plastids originated.

**Figure 1:**
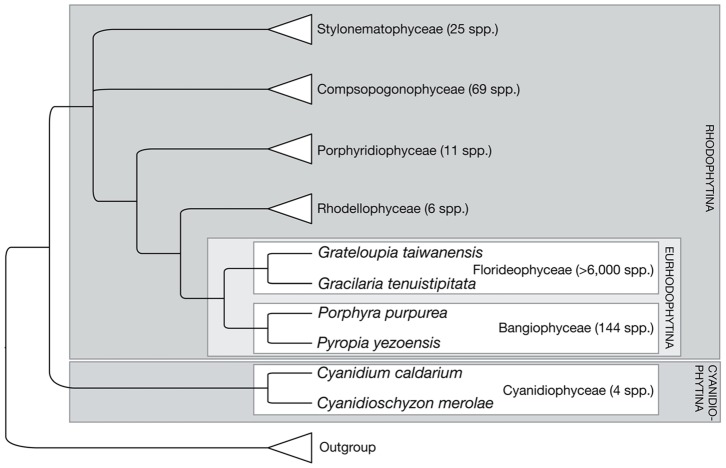
Phylogeny of Rhodophyta, adapted from Yoon *et al.* [6]. Numbers of species are from AlgaeBase [1].

Molecular phylogenetic analysis has divided the red algae into seven classes [6] [7]. This phylogeny is given in Figure 1. Almost all red algal species – over 6,000– belong to the class Florideophyceae, which is most closely related to the class Bangiophyceae (∼150 species [1]). These two classes have been grouped in the subphylum Eurhodophytina. The most anciently diverged of the classes, the Cyanidiophyceae, consists of very few species divided into three genera of extremophilic unicellular algae known to inhabit acidic hot springs. Five red algal plastid genomes have been published thus far, including representatives of these three classes: *Gracilaria tenuistipitata* var. *liui* Zhang & Xia (Florideophyceae); *Porphyra purpurea* (Roth) C.Agardh and *Pyropia yezoensis* (Ueda) M.S.Hwang & H.G.Choi (Bangiophyceae); and *Cyanidium caldarium* (Tilden) Geitler and *Cyanidioschyzon merolae* P.De Luca, R.Taddei & L.Varano strain 10D (Cyanidiophyceae). Because almost all known red algal diversity is found in the Florideophyceae, the plastid genome sequence of a single species (*G. tenuistipitata* var. *liui*) is clearly insufficient information to understand the whole spectrum of characteristics that are shared by florideophycean plastids. A thorough understanding of present-day red algal plastids, with sufficient coverage across the red algal tree of life, can help demonstrate the characteristics of ancestral red algae and their plastids, which would have been the source of the secondary endosymbiotic plastids of the brown algal lineages.

The florideophycean genus *Grateloupia* C. Agardh contains around 90 species [1] of benthic macroalgae that are distributed in warm temperate to tropical waters worldwide. Some species of *Grateloupia* are known invasive species. *Grateloupia taiwanensis* S.-M.Lin & H.-Y. Liang was first described in 2008 by Lin *et* *al.* [8] but it has since been recorded in the Gulf of Mexico [9].The genus is currently being split into several genera based on combined molecular and morphological analysis [10], and it is possible that *G. taiwanensis* will be placed into a new genus.

*Grateloupia* belongs to the order Halymeniales, whereas *Gracilaria tenuistipitata* var. *liui* is in the order Gracilariales. Both orders are classified in the subclass Rhodymeniophycidae, but their phylogenetic relationships within the subclass are unresolved, due to consistent ambiguity in the phylogenetic position of Gracilariales [11] [12] [13]. Comparisons between the plastid genomes of *Gracilaria tenuistipitata* and *Grateloupia taiwanensis* will establish a basis for contrasting the common characteristics of the plastid in Florideophyceae with those of the other classes, as well as comparing the plastids of Rhodymeniophycidae with the other subclasses of Florideophyceae, which have yet to be published.

## Materials and Methods

An individual of *Grateloupia taiwanensis* from Orange Beach, AL, USA, which was collected in a previous study [9] was selected for genome sequencing. DNA was extracted from the field-collected sample using the QIAGEN DNEasy Plant Mini Kit (QIAGEN, Valencia, CA, USA) following the manufacturer's instructions. The sequencing library was prepared using the Nextera DNA Sample Prep Kit (Illumina, San Diego, CA, USA) per the manufacturer's protocol and sequenced on one-half lane of an Illumina Genome Analyzer IIX using the TruSeq SBS Kit v5 (Illumina) in a 150×150 bp paired-end run. The data were adapter- and quality-trimmed (error threshold  = 0.05, n ambiguities  = 2) using CLC Genomics Workbench (CLC Bio, Aarhus, Denmark) prior to de-novo assembly with same (automatic bubble size, minimum contig length  = 100 bp). The raw reads were then mapped to the assembly contigs (similarity  = 90%, length fraction  = 75%), and regions with no evidence of short-read data were removed. The resulting assembly included one large contig 191,270 bp in size, which was determined to be the plastid genome by several criteria: (1) BLAST searches [14] of commonly known plastid genes against the entire assembly produced hits on this contig with significant *e-*values (*e*≤10^−20^); (2) a genome size of 191,270 bp is congruent with the sizes of other red algal plastid genomes, which range from 150 to 191 kbp [15]; (3) because each cell contains many plastids and therefore many copies of the plastid genome, it follows that cpDNA will be relatively over-represented in the short sequence reads.

The *G. taiwanensis* plastid genome was imported to Geneious (Geneious version 5.1.7; available from http://www.geneious.com/) and set to circular topology. Using the Geneious ORF Finder and the standard genetic code, the start codons ATG and GTG, and a minimum length of 90 bp, the genome contained 768 ORFs. Preliminary annotation was performed using DOGMA [16] with an *e-*value cutoff of 10^−20^ for BLAST hits. After alignments for each gene, these were checked manually and the corresponding ORF in the genome sequence was annotated. The remaining ORFs were translated using the standard genetic code and submitted to phmmer (http://hmmer.janelia.org/), searching against the UniProtKB database (http://www.uniprot.org). After including the additional start codon TTG, any ORFs occurring outside any annotation were searched for functional domains using the InterProScan Geneious plugin version 1.0.5 [17]. Annotations for those ORFs with putative functional domains were included in the genome.

To determine tRNA sequences, the plastid genome was submitted to the tRNAscan-SE version 1.2.1 server [18] [19]. The genome was searched with default settings using the “Mito/Chloroplast” model. To determine rRNA sequences, a set of known plastid rRNA sequences was extracted from the *Gracilaria tenuistipitata* var. *liui* genome and used as a query sequence to search the *G. taiwanensis* genome using BLAST. A search for tmRNA sequences was performed using BRUCE v1.0 [20]. The genome was visualized using GenomeVx [21] and edited using Adobe Illustrator CS2 (http://www.adobe.com/products/illustrator.html).

The five published red algal plastid genomes, with annotations, were downloaded from GenBank. Gene names were checked with the preferred name in UniProtKB and revised in order to make the most accurate comparisons between genomes. In situations where one gene had multiple names, if all were orthologous according to BLAST (*e* ≤10^−10^) against UniProtKB, the name used by the majority of species was used. Names of known and putative protein-coding genes (*i.e.,* excluding tRNAs or rRNAs) were extracted from the genomes, and the sets were compared using VENNTURE [22]. Genes found to be missing from a certain species or group of species were checked using BLAST in order to ensure that this gene is not present. For structure and arrangement comparisons, the genomes were aligned using the Mauve Genome Alignment version 2.2.0 [23] Geneious plugin using the progressiveMauve algorithm [24] and default settings. To aid in visualization, we designated the beginning of the *rbc*L marker as position 1 in each genome.

## Results

### The *Grateloupia taiwanensis* plastid genome

**Figure 2:**
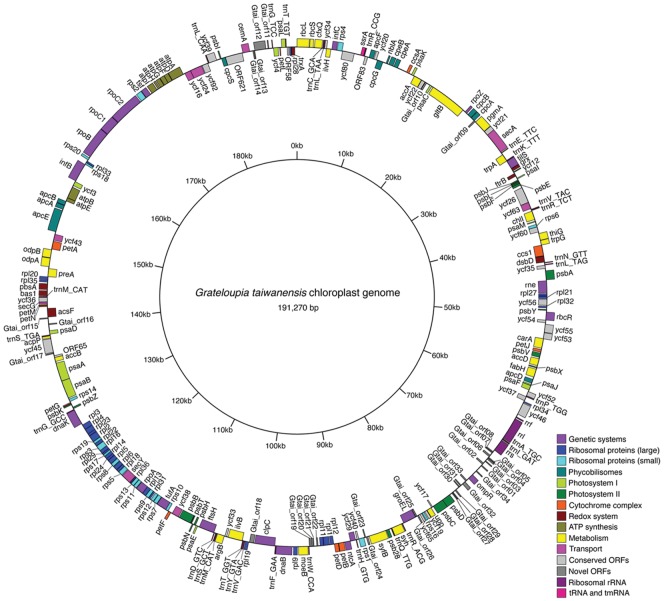
The *Grateloupia taiwanensis* plastid genome. Colors indicate different gene classifications, as listed in Table 2.

The 191,270 bp plastid genome (Figure 2) includes 233 ORFs identified as protein-coding genes, of which 35 are found only in *G. taiwanensis* and not in the other red algae examined in this study. Additionally, it contains 29 tRNA sequences, 3 rRNA sequences, and 1 tmRNA sequence (Table 1). The rRNA operon is not repeated. The tmRNA sequence appears to be homologous to the *ssr*A tmRNA of *Gracilaria tenuistipitata* var. *liui.* The GC-content of the *G. taiwanensis* plastid genome is 30 1). The proportion of intergenic space in *G. taiwanensis* was 18.1%, which is comparable to the other Eurhodophytina and higher than the Cyanidiophyceae (Table 1). The sequence was deposited in GenBank (accession number KC894740).

**Table 1:** Characteristics of red algal plastid genomes analyzed in this study.

	Florideophyceae		Bangiophyceae		Cyanidiophyceae	
	*Grateloupia taiwanensis*	*Gracilaria tenuistipitata* var. *liui*	*Porphyra purpurea*	*Pyropia yezoensis*	*Cyanidioschyzon merolae* strain 10D	*Cyanidium caldarium*
**General characteristics**						
Size (bp)	191,270	183,883	191,028	191,952	149,987	164,921
G+C (%)	30.6	29.2	33.0	33.1	37.6	32.7
Intergenic space (%)	18.1	15.5	15.3	15.9	9.3	10.5
Protein-coding genes						
Number of protein-coding genes	234	204	207	207	193	199
Unique gene annotations	35	13	0	0	4	15
Number of ribosomal proteins	47	47	47	46	46	45
**Start codon usage** (**%**)						
ATG	87.6	89.7	91.8	92.3	97.9	98.5
GTG	6.0	2.0	5.8	5.3	2.1	1.0
TTG	6.4	7.8	2.4	1.4	–	0.5
others	–	0.5	–	1.0	–	–
**RNAs**						
Number of tRNAs	29	29	37	38	31	30
Number of rRNA operons	1	1	2	2	1	1
GenBank accession	KC894740	AY673996	PPU38804	AP006715	AB002583	AF022186

Intergenic space is defined as any portion of the genome that does not bear a gene or RNA annotation.

### Gene content

All of the plastid genomes considered in this study share a set of 140 protein-coding genes, and an additional 21 genes are shared among the Eurhodophytina (Table 2). Five additional genes are shared only between *G. taiwanensis* and *G. tenuistipitata* var. *liui*. In total, 167 of the protein-coding genes found in the plastid of *G. taiwanensis* are shared with *G.tenuistipitata* var. *liui*. Of the 35 putative genes found only in *G. taiwanensis*, one is a gene for glutaredoxin (*grx*). This *grx* gene is 104 aa in length and is most similar to that of the cyanobacterium *Arthrospira platensis* (UniProt blastx, match length 107 aa, 78.0% positives, e = 8.0×10^−38^). The remaining 34 genes are unique ORFs with functional domains indicated by InterProScan (see Table S1 for annotations). *G. taiwanensis* and *G. tenuistipitata* var. *liui* share the same 29 plastid tRNA genes (Table 3). *Porphyra purpurea* and *Pyropia yezoensis* contain more tRNA genes than the others, with 37 and 38, respectively; two tRNA genes – *trn*I(GAT) and *trn*A(TGC) – occur inside the repeated rRNA operon. In terms of tRNA gene content, the Florideophyceae and Cyanidiophyceae are more similar to each other than to the Bangiophyceae.

**Table 2:** List of genes in the *Grateloupia taiwanensis* plastid genome (233 total).

*Classification*	*Number*	*Genes*							
**Genetic systems**									
Maintenance	2	**dnaB**	rne						
RNA polymerase	5	**rpoA**	**rpoB**	rpoC1	rpoC2	rpoZ			
Transcription factors	4	ntcA	ompR	rbcR	ycf29				
Translation	4	infB	**infC**	**tsf**	**tufA**				
**Ribosomal proteins**									
* Large subunit*	28	**rpl1**	**rpl2**	**rpl3**	**rpl4**	**rpl5**	**rpl6**	rpl9	**rpl11**
		**rpl12**	**rpl13**	**rpl14**	**rpl16**	**rpl18**	**rpl19**	**rpl20**	**rpl21**
		**rpl22**	**rpl23**	**rpl24**	**rpl27**	**rpl28**	**rpl29**	**rpl31**	**rpl32**
		**rpl33**	**rpl34**	**rpl35**	**rpl36**				
* Small subunit*	19	rps1	**rps2**	**rps3**	**rps4**	**rps5**	**rps6**	**rps7**	**rps8**
		**rps9**	**rps10**	**rps11**	**rps12**	**rps13**	**rps14**	**rps16**	**rps17**
		**rps18**	**rps19**	**rps20**					
tRNA processing	1	tilS							
Protein quality control	4	**clpC**	**dnaK**	**ftsH**	**groEL**				
**Photosystems**									
Phycobilisomes	12	**apcA**	**apcB**	**apcD**	**apcE**	**apcF**	**cpcA**	**cpcB**	**cpcG**
		cpcS	cpeA	cpeB	nblA				
Photosystem I	13	**psaA**	**psaB**	**psaC**	**psaD**	**psaE**	**psaF**	**psaI**	**psaJ**
		**psaK**	**psaL**	**psaM**	**ycf3**	**ycf4**			
Photosystem II	19	**psbA**	**psbB**	**psbC**	**psbD**	**psbE**	**psbF**	**psbH**	**psbI**
		**psbJ**	**psbK**	**psbL**	**psbN**	**psbT**	**psbV**	**psbX**	**psbY**
		psbZ	psb28	**ycf12**					
Cytochrome complex	11	ccs1	ccsA	**petA**	**petB**	**petD**	**petF**	**petG**	**petJ**
		petL	petM	petN					
Redox system	7	acsF	bas1	dsbD	ftrB	grx	pbsA	trxA	acsF
**ATP synthesis**									
ATP synthase	8	**atpA**	**atpB**	**atpD**	**atpE**	**atpF**	**atpG**	**atpH**	**atpI**
**Metabolism**									
Carbohydrates	6	**cfxQ**	**odpA**	**odpB**	pgmA	**rbcL**	**rbcS**	**cfxQ**	**odpA**
Lipids	5	**accA**	**accB**	**accD**	**acpP**	fabH	**accA**	**accB**	**accD**
Nucleotides	2	**carA**	upp						
Amino acids	8	**argB**	**gltB**	**lab**	**ilvH**	syfB	syh	**trpA**	**trpG**
Cofactors	4	**chlI**	moeB	**preA**	**thiG**	**chlI**	moeB	**preA**	**thiG**
**Transport**									
Transport	9	cemA	**secA**	secG	**secY**	**ycf16**	**ycf24**	ycf38	ycf43
		ycf63							
**Unknown**									
Conserved ORFs	28	ORF58	*ORF65*	*ORF83*	ORF621	**ycf17**	**ycf19**	**ycf20**	ycf21
		ycf22	ycf26	ycf33	ycf34	ycf35	ycf36	ycf37	**ycf39**
		**ycf40**	ycf45	ycf46	**ycf52**	**ycf53**	**ycf54**	**ycf55**	ycf56
		**ycf60**	**ycf65**	**ycf80**	ycf92				
Unique ORFs	34	Gtai_orf01, Gtai_orf02, …, Gtai_orf34							

Genes in bold are shared among all red algal plastids (140 total). Genes underlined are shared among Eurhodophytina (21 total). Genes italicized are shared among Florideophyceae (5 total). Categories for classification follow Ohta *et al.* [30].

**Table 3:** tRNA sequences present in red algal plastid genomes.

	trnA (GGC)	trnA (TGC)	trnC (GCA)	trnD (GTC)	trnE (TTC)	trnF (GAA)	trnG (GCC)	trnG (TCC)	trnH (GTG)	trnI(GAT)	trnK (TTT)	trnL(CAA)	trnL(GAG)	trnL(TAA)	trnL(TAG)	trnM(CAT)	trnN(GTT)	trnP(TGG)	trnQ(TTG)	trnR(ACG)	trnR(CCG)	trnR(CCT)	trnR(TCT)	trnS(CGA)	trnS(GCT)	trnS(GGA)	trnS(TGA)	trnT(GGT)	trnT(TGT)	trnV(GAC)	trnV(TAC)	trnW(CCA)	trnY(GTA)
*Cyanidiumcaldarium*		1	1	1	1	1	1	1	1	1	1	1	1	1	1	3	1	1	1	1	1		1		1		1	1	1	1	1	1	1
*Cyanidioschyzonmerolae*		1	1	1	1	1	1	1	1	1	1	1	1	1	1	3	1	1	1	1			1		1	1	1	1	1	1	1	1	1
*Porphyrapurpurea*	1	2	1	1	1	1	1	1	1	2	1	1	1	1	1	3	1	1	1	1	1	1	1	1	1	1	1	1	1	1	1	1	1
*Pyropiayezoensis*	1	2	1	1	1	1	1	1	1	2	1	1	1	1	2	3	1	1	1	1	1	1	1	1	1	1	1	1	1	1	1	1	1
*Gracilariatenuistipitata*var. *liui*		1	1	1	1	1	1	1	1	1	1	1		1	1	2	1	1	1	1	1		1		1		1	1	1	1	1	1	1
*Grateloupiataiwanensis*		1	1	1	1	1	1	1	1	1	1	1		1	1	2	1	1	1	1	1		1		1		1	1	1	1	1	1	1

Anticodon sequence is given in 3′–5′ direction.

### Plastid genome rearrangements

**Figure 3:**
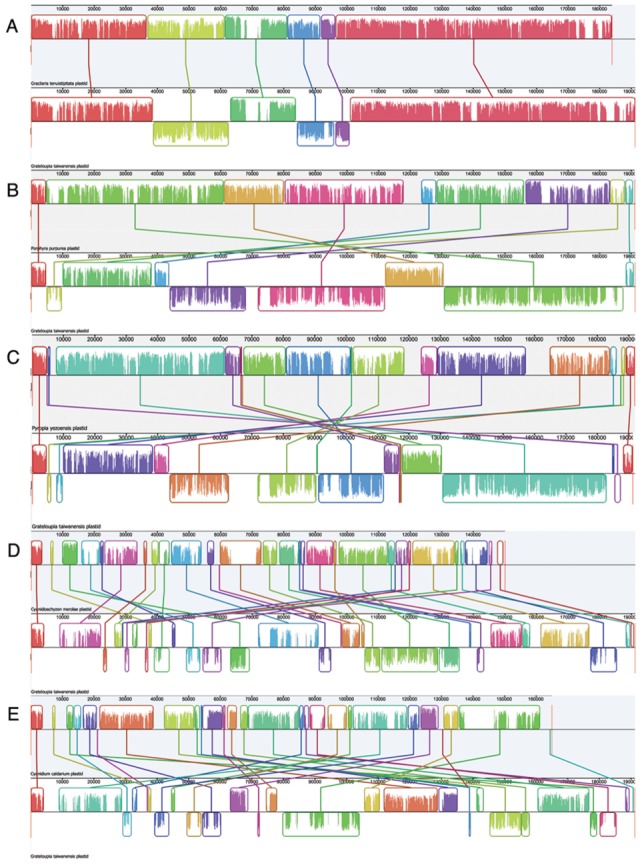
Mauve genome alignments of linearized plastid genomes, with *G.* *taiwanensis* set as reference. Corresponding colored boxes indicate locally collinear blocks (LCBs), which represent homologous gene clusters. LCBs below the horizontal line in the second genome indicate reversals. Heights of vertical bars within LCBs indicate relative sequence conservation at that position. A: *G. taiwanensis* and *Gracilaria tenuistipitata*; B: *G. taiwanensis* and *Porphyra purpurea*; C: *G. taiwanensis* and *Pyropia yezoensis*; D: *G. taiwanensis* and *Cyanidioschyzon merolae*; E: *G. taiwanensis* and *Cyanidium caldarium*.

Pairwise Mauve genome alignments for *G. taiwanensis* along with each other five plastid genomes used in this study are given in Figure 3. We calculated the double-cut-and-join (DCJ) genome distance, indicative of the number of rearrangements that have taken place between two genomes. The alignment of *G. taiwanensis* and *Gracilaria tenuistipitata* var. *liui* shows a DCJ distance of 3; *G. taiwanensis* and *Porphyra purpurea*, 4; *G. taiwanensis* and *Pyropia yezoensis*, 8; *G. taiwanensis* and *Cyanidioschyzon merolae*, 20; *G. taiwanensis* and *Cyanidium caldarium*, 21.

## Discussion

The plastid genome of *G. taiwanensis* is similar to that of *G. tenuistipitata* var. *liui* in terms of size, GC%, gene content, and overall structure. However, there are several notable differences; *G. taiwanensis* contains 67 putative protein-coding genes not present in *G. tenuistipitata* var. *liui*, including 32 previously named genes and 34 novel ORFs. When additional plastid genome sequences for Florideophyceae become available, it is possible that many of these novel ORFs will be found in other red algae.

The results of the current study are generally consistent with the phylogeny of Rhodophyta proposed by Yoon *et al*. [7]. Unlike in *Porphyra purpure*a and *Porphyra yezoensis*, in which the rRNA operon is repeated directly, *G. taiwanensis* has only one rRNA operon. This is consistent with the hypothesis of Hagopian *et al.* [25] that the repeated rRNA operon was lost separately in the Cyanidiophyceae and the Florideophyceae. A similar pattern arose in the tRNA genes in Cyanidiophyceae and Florideophyceae. The reason for this is unclear, but because it is commonly accepted that the Cyanidiophyceae is the sister group to the rest of the red algae, we suggest that this is an example of convergent gene loss.

As expected, our analyses show that pairs of plastid genomes of red algae found in the same taxonomic class demonstrate the most structural and functional similarity (*Cyanidioschyzon/Cyanidium*, *Porphyra/Pyropia*, and *Grateloupia/Gracilaria*), which decreases withthe degree of relatedness. The presence of 140 “core” plastid genes reflects high conservation in the plastids of red algae, compared to green algal plastids, which show much more variability in genome size, GC%, and other attributes [26]. Despite their similar sizes, red algal plastid genomes contain many more genes than green algal genomes, and the genes are packed tightly together with much less intergenic sequence. Thus far, *G. taiwanensis* shows the most intergenic sequence of any red algal plastid (18.1%), but this value is relatively low compared to those of green algal plastids.

As more and more genomes are annotated and published, comparative genomics of primary and secondary plastids will provide new insights into the pattern and process of endosymbiosis, especially in those lineages with red-derived plastids. The genes shared among all red algal plastids are likely to be essential for plastid function in Rhodophyta and offer a useful starting point for future annotation of plastid genomes. Several previous studies focused on red-derived plastids [27] [28] [29] have shown the potential of plastid genome research in answering unresolved questions in the history of these lineages. For these reasons, red algal plastid genomes remain a highly interesting subject for research. Forthcoming sequence data will advance our understanding of the evolution of the red algal plastid.

## Supporting Information

Table S1 **Novel ORFs found in the** ***G. taiwanensis*** **plastid genome.**
(DOCX)
