# Supplementary material for: The Plastid Genome of the Red Macroalga Grateloupia taiwanensis (Halymeniaceae)
Source: PLoS One. 2013 Jul 19;8(7):e68246. doi: 10.1371/journal.pone.0068246 (PMC3716797; doi:10.1371/journal.pone.0068246)
Supplement: Table S1 — Novel ORFs found in the G. taiwanensis plastid genome. (DOCX) [file pone.0068246.s001.docx]

| *ORF name* | *Strand* | *Start position* | *Stop position* | *Length (aa)* | *InterPro terms and positions (aa)* |
| --- | --- | --- | --- | --- | --- |
| Gtai_orf01 | + | 70306 | 70428 | 40 | Signal_peptide (1-38); transmembrane_regions (19-39) |
| Gtai_orf02 | - | 70176 | 69994 | 60 | Signal_peptide (1-26); transmembrane_regions (28-46) |
| Gtai_orf03 | + | 69560 | 69697 | 35 | Signal_peptide (1-16) |
| Gtai_orf04 | + | 69168 | 69326 | 52 | Signal_peptide (1-42); transmembrane_regions (22-40) |
| Gtai_orf05 | + | 69026 | 69133 | 35 | Signal_peptide (1-20) |
| Gtai_orf06 | - | 68933 | 68727 | 68 | Signal_peptide (1-36) |
| Gtai_orf07 | - | 68371 | 68234 | 45 | Signal_peptide (1-42); transmembrane_regions (21-41) |
| Gtai_orf08 | - | 68273 | 68169 | 34 | Signal_peptide (1-32); transmembrane_regions (12-32) |
| Gtai_orf09 | - | 24162 | 23983 | 59 | Signal_peptide (1-48); transmembrane_regions (37-57) |
| Gtai_orf10 | - | 16593 | 16402 | 63 | Signal_peptide (1-17); transmembrane_regions (34-52) |
| Gtai_orf11 | + | 187730 | 187831 | 33 | Signal_peptide (1-28) |
| Gtai_orf12 | + | 186114 | 187463 | 449 | RVT_N (12-94) |
| Gtai_orf13 | - | 186082 | 185957 | 41 | Signal_peptide (1-26) |
| Gtai_orf14 | - | 185982 | 185860 | 40 | Signal_peptide (1-26) |
| Gtai_orf15 | + | 140795 | 140905 | 36 | Signal_peptide (1-22) |
| Gtai_orf16 | - | 140163 | 140044 | 39 | Signal_peptide (1-23) |
| Gtai_orf17 | + | 136471 | 136569 | 32 | Signal_peptide (1-26) |
| Gtai_orf18 | - | 101463 | 100945 | 172 | Transmembrane_regions (43-63 and 84-104) |
| Gtai_orf19 | - | 94129 | 93992 | 45 | Signal_peptide (1-33); transmembrane_regions (15-37) |
| Gtai_orf20 | - | 93893 | 93756 | 45 | Signal_peptide (1-19) |
| Gtai_orf21 | - | 93659 | 93558 | 33 | Transmembrane_regions (14-32) |
| Gtai_orf22 | - | 93209 | 93072 | 45 | Signal_peptide (1-15); transmembrane_regions (5-25) |
| Gtai_orf23 | - | 87274 | 87164 | 36 | Signal_peptide (1-16); transmembrane_regions (9-29) |
| Gtai_orf24 | + | 86893 | 87087 | 64 | Signal_peptide (1-21); transmembrane_regions (23-43) |
| Gtai_orf25 | - | 80539 | 80387 | 50 | Transmembrane_regions (20-40) |
| Gtai_orf26 | + | 80181 | 80324 | 47 | Transmembrane_regions (14-34) |
| Gtai_orf27 | + | 75209 | 75304 | 31 | Signal_peptide (1-27) |
| Gtai_orf28 | + | 75105 | 75194 | 29 | Signal_peptide (1-23); transmembrane_regions (10-28) |
| Gtai_orf29 | + | 74647 | 74787 | 46 | Signal_peptide (1-32) |
| Gtai_orf30 | - | 74186 | 74079 | 35 | Signal_peptide (1-22); transmembrane_regions (14-34) |
| Gtai_orf31 | - | 73946 | 73824 | 40 | Signal_peptide (1-30); transmembrane_regions (15-37) |
| Gtai_orf32 | + | 73194 | 73592 | 132 | DUF1368 (17-121) |
| Gtai_orf33 | - | 73063 | 72899 | 54 | Signal_peptide (1-21); transmembrane_regions (4-22 and 31-51) |
| Gtai_orf34 | + | 71228 | 71341 | 37 | Signal_peptide (1-29); transmembrane_regions (15-35) |

List of novel ORFs in the *Grateloupia taiwanensis* plastid genome. Start codons include ATG, GTG, and TTG; minimum length = 90 nucleotides. Positions in sequence given with position 1 on the “+” strand defined as the beginning of the *rbc*L gene. Calculated amino acid length does not include the stop codon.
